# Evolution of Curative Therapies For Atrial Fibrillation

**Published:** 2004-01-01

**Authors:** Atul Khasnis, Srikar Veerareddy, Krit Jongnarangsin, John H Ip, George S Abela, Ranjan K Thakur

**Affiliations:** Arrhythmia Service, Thoracic and Cardiovascular Institute, E.W. Sparrow Heart Center, Michigan State University, Lansing, Michigna, USA

## Introduction

Atrial fibrillation (AF) is one of the most common arrhythmias seen in clinical practice. The incidence of AF increases with age and is seen in up to 8.9% of individuals greater than 80 years of age [1]. Although many treatment modalities are available for AF, curative therapy has recently become possible for some patients with atrial fibrillaton. This is partly due to the complexity and incomplete understanding of this arrhythmia. Pharmacological therapies for the management of AF have many limitations such as side effects, cost, inconvenience, breakthrough arrhythmias, drug-drug interactions and continued need for anticoagulation. An improved understanding of the pathophysiology and electrophysiologic basis of AF coupled with the availability of better mapping techniques as well as advances in catheter technology offer promise for curative treatment. This article will briefly review the understanding of the mechanisms of AF and then the evolution of catheter techniques for control and elimination of AF.

## Mechanisms of AF

The understanding of mechanisms underlying the initiation and maintenance of AF has evolved over the last many decades. The earliest concept of reentry was proposed by Winterberg in 1906 [2] and Lewis in 1912 advocated that rapid focal activity from one or more centers accounted for AF [3]. Mines in 1913 showing that the mechanism of reentry was an impulse circling a large anatomical obstacle [4]. Scherf in 1947 revived the theory of focal trigger in AF [5]. The work of Moe in 1960s supported the theory of randomly propagating multiple wavelets as the main mechanism underlying AF [6]. The reentrant wavelet hypothesis required the concept of “wavelength” of the arrhythmia circuit to be introduced. The minimum circuit wavelength as defined by the tachycardia wavelength is the product of the tissue refractory period and wavefront conduction velocity. Any circuit wavelength smaller than the minimum will cause the “head” of the wavefront to catch up with its “tail” thus terminating the reentry. In the 1970s, Allessie introduced the concept of “leading circle reentry”. In a goat model of AF, he demonstrated that the average circuit diameter was 20 - 30 mm and that a minimum of 5-8 random wavelets were required to sustain AF at any given time. Two types of reentry were seen during the study: “leading circle” reentry and “random” reentry [7]. All these theories of random reentry wavelets explained the sustenance of AF once it occurred, but an explanation of its initiation was also warranted. Allessie et al. offered several possible explanations: a “stable background circuit” capable of initiating new AF when the earlier episode dies out, abnormal focal trigger sites in the atria and the possibility of an echo beat from the AV node or from an accessory pathway. The present understanding is that AF requires a “critical atrial mass” needed to maintain the arrhythmia and that there is a critical rate above which organized atrial activity cannot continue [8]. Thus, at a certain rate, organized atrial activity can disintegrate into atrial fibrillation provided the critical tissue mass is available to sustain it. Recent studies in isolated human atrial preparations showed that a single meandering functional reentrant wavefront produced atrial fibrillation [9]. Recent work by Jalife and colleagues questions the randomness of atrial activity in atrial fibrillation [10]. Their study suggests the presence of a possible “mother circuit” that serves as a periodic background focus; the presence of anatomical obstacles (scar, orifices) serve to break up the wavefront from the “mother circuit” into multiple wavelets that spread in various directions. Wu et al. have proposed the role of pectinate muscles as obstacles that break the activation wave thus promoting reentry. They may also serve as an anchoring site for the wave leading to rotor like activity [11]. The likelihood that focal activation plays some role in atrial fibrillation is now well accepted. Haissaguerre et al. reported arrhythmogenicity of the pulmonary veins as possible focal triggers in some cases of atrial fibrillation [12]. The myocardial sleeves that extend from the left atrium onto the pulmonary veins appear to be the pathological correlate of the arrhythmogenic focus. Thus there appears to be a role for both reentry and focal activity in the electrophysiology of AF. The question of randomness of AF is also not settled at present. Treatment modalities aim at elimination of both mechanisms underlying AF. The following sections discuss how the above discoveries have channeled catheter therapy for AF.

## Mapping AF

The discussion of evolution of catheter techniques would be impossible without inclusion of the development of mapping techniques [13]. However a detailed description is beyond the scope of this article. There has been a tremendous impact of mapping technology on diagnostic and therapeutic electrophysiology. The mapping of AF has helped clarify its mechanism and localize possible anatomical sites for effective radiofrequency ablation. Conventionally, this has been done by careful correlation of 12-lead surface ECG with intracardiac data detected by catheters lying in contact with the endocardium in various cardiac chambers. However, these methods do not cover a vast area of the endocardial surface and spot-by-spot maneuvering of the catheter is required to trace a focus and determine the sequence of impulse spread to surrounding areas. Another drawback of this technique is inability to identify several sites and then return to the most optimal site. Hence it is important to achieve a three dimensional orientation of the focus and activation sequence to be able to localize therapy. Electroanatomic or CARTO™ mapping is a nonfluoroscopic mapping system that uses magnetic technology to accurately determine the location and orientation of the mapping and ablation catheter while simultaneously recording local electrograms from the catheter tip. The 3-D geometry of the mapped chamber is reconstructed in real-time and the optimal sites for ablation are analyzed. The timing of the reference electrogram is used to determine the activation timing in relation to the acquired points and therefore collection of data during the corresponding parts of the cardiac cycle is vital to the performance of the system. Noncontact Mapping using the EnSite3000™ (Endocardial Solutions, St Paul, MN) mapping system consisting of balloon or multielectrode array (MEA) has been studied extensively. This technique is based on the principle that endocardial activation produces a chamber voltage field, which obeys Laplace’s equation. Noncontact intracavitary electrodes are used to detect the potential field on the endocardial surface. An inverse Laplace’s equation is used to improve accurate and stable reconstructions of the recorded potentials. The activation points are displayed as computed electrograms or isopotential maps. A three-step process of establishing geometry, identifying the area of interest and navigating the ablation catheter to this area is used to map and treat arrhythmias. Focal atrial fibrillation arising from both right atrium and pulmonary veins has been successfully ablated using this system [14]. Other techniques used include the Basket and amplification technique. In order to characterize the local electrical activity of the heart, a "basket" catheter is used with 8 splines carrying 8 electrodes each (EPT, Boston Scientific). The electrodes are coupled two by two to achieve bipolar recordings thus producing 32 bipolar recordings. Each electrode couple is then amplified and filtered separately for every channel (CardioLab System, Prucka Engineering, Huston, USA). Amplification gain is normally set between 500 and 2500 to achieve best signal reading; filters are set between 30 and 500 Hz. The signals are then sampled at 1 kHz and stored on magneto-optical device. Intracardiac echocardiography (ICE) can be a valuable tool in localizing anatomical areas for ablation. It allows for assessment of wall contact of ablation catheters for creation of long linear lesions for catheter ablative treatment of atrial fibrillation. Epstein et al. demonstrated the superiority of ICE over fluoroscopy in treating atrial fibrillation [15]. Inverse electrocardiography, an established formulation is the imaging of the activation time map on the entire surface of the heart from ECG mapping data [16,17]. This enables reconstruction of unifocal, multifocal and more distributed activation patterns. It can distinguish between areas with early and late activation. This novel approach is presently under clinical evaluation and development. A new non-invasive technique for the characterization of time-dependent spectral properties of atrial fibrillation in the surface ECG using the Wigner-Ville distribution for time-frequency analysis and the cross Wigner-Ville to compute trends describing instantaneous frequency of the atrial activity has also been described. Preliminary results indicate that short-term variations exist in the fibrillation cycle lengths and that the variations can exhibit similar behavior in the leads V1-V3 [18]. Magnetic resonance imaging has also shown promise in demonstrating pulmonary venous anatomy which is central to the technique of radiofrequency ablation of focal AF [19]. The last three techniques are non-invasive.

## Radiofrequency catheter ablation of AF

Treatment of AF using radiofrequency techniques can be curative or palliative. The curative strategies will be discussed in this review. The palliative procedures will be briefly be touched upon.

### Curative techniques: The Maze Operation - ablation of reentrant AF

#### Surgical Maze procedure

The understanding of the electrophysiology of AF as consisting of multiple random circulating reentrant wavelets was the basis for the development of the Maze operation. The originally devised Maze operation was an open heart surgical procedure that involved creating multiple surgical incisions (the maze) in both atria aimed at interruption of reentry of random wavefronts while permitting the impulse to travel in a channeled direction to the AV node. This operation underwent serial modifications beginning with the left atrial isolation procedure in 1980 and progressing to the Maze III operation which resulted in higher incidence of postoperative sinus rhythm, improved long term sinus node function, decreased need for pacing, less arrhythmia recurrence and improved long-term atrial transport function. The Maze III procedure can now be successfully performed as a minimally invasive procedure through a submammary incision. The epicardial application of radiofrequency energy for production of lesions without cardiopulmonary bypass was an attempt at reducing morbidity and complications associated with the Maze procedure.

#### Maze procedure using radiofrequency energy

The use of radiofrequency catheter techniques for selective right or left atrial modification involves the creation of linear transmural atrial lesions by endocardial radiofrequency current application. The right atrial modification technique has met with good success in cases where the AF is triggered by a short run of atrial flutter or in cases when a single macro-reentrant circuit is located in the right atrium and has an unstable cycle length. The site of application of radiofrequency current in the right atrium varies from a single midseptal line to the region of the Bachmann’s bundle to the posterior intercaval right atrium in experimental models. On the other hand, left atrial modification has not been rewarding because of faulty technique and increased risk of stroke. In experimental studies, left posterior atrial modification with rapid atrial pacing and left atrial lines (in mitral regurgitation model) has been reported successful. The left atrium is now frequently the target for curative AF ablation since the reporting of pulmonary veins as triggers for AF (pulmonary vein ablation is discussed later in the article). A number of early investigators reported success with use of radiofrequency energy to create long linear lesions in both animals and humans. Elvan et al. reported successful cure of AF by application of radiofrequency current to five epicardial sites and one endocardial site (coronary sinus) in a canine model of AF induced by burst atrial pacing [20]. The problem with earlier catheter design was the lack of good tissue contact leading to the production of discontinuous lesions which could themselves be proarrhythmic. Development in catheter technology and mapping has overcome this shortcoming. Olgin et al. reported the use of intracardiac echocardiography in the pig heart for anatomical localization aiding placement of intraatrial catheters for ablation [21]. This also helped achieve good tissue contact and successful transmural ablation as confirmed by histologic evaluation. Kalman et al. showed that the production of linear atrial lesions reduced the energy requirement for cardioversion of AF and in some cases to its termination [22]. Swartz et al. reported creating linear atrial lesions in humans with chronic AF using the drag technique [23]. The main disadvantages were long procedure and fluoroscopy time. In 1997, Patwardhan et al [24]. reported success of the Maze procedure using radiofrequency bipolar coagulation as a means of energy to produce atrial lesions. Their study population included patients with rheumatic heart disease and atrial fibrillation. They used radiofrequency microbipolar coagulation to produce conduction blocks along incision lines in keeping with the surgical Maze III procedure as an adjunct to valve surgery in 18 patients in atrial fibrillation undergoing surgery for rheumatic valvular disease. The control population consisted of those 26 patients who underwent corrective valve surgery alone. The energy was delivered by a bayonet type bipolar forceps with an active tip length of 7 mm drawing current from a microbipolar port of Valleylab Force 4 electrosurgical unit (Valleylab, Boulder, CO). A 3-mm retinal handheld cryoprobe using nitrous oxide gas was used for cryoablation. 15/18 patients in the MAZE group were followed-up. 12/15  (80%) patients converted to normal sinus rhythm. Pulsed wave Doppler evaluation at follow-up showed return of atrial transport function, presence of a wave in all these patients in tricuspid valve flow and in nine (75%) patients in mitral valve flow. The procedure took 11.62 +/- 3.86 min of elective cardioplegic arrest time for ablation line production in the left atrium and 18.71 +/- 4.25 min of cardiopulmonary bypass time during reperfusion for ablation line production in the right atrium. Only 1/23 patients in the control (4.3%) converted to normal sinus rhythm. The investigators concluded that their modification considerably shortened the procedure for the Maze procedure and was effective in restoring normal sinus rhythm in 80% of the patients. Haissaguerre et al. reported successful ablation of AF using a 7-F specially designed 14-polar catheter with interelectrode distance of 3 mm to create linear lesions in the right atrium using radiofrequency energy [25]. Calkins et al. performed a Maze-like procedure using the Guidant Heart Rhythm Technologies Linear Ablation System to create long transmural lesions [26]. The unique features of this system included the availability of different pre-shaped multi-electrode steerable ablation catheters, the use of multi-phased radiofrequency energy and control of RF output by varying the duty cycle. Fourteen out of 15 patients enrolled in this study had an acute successful outcome.

#### Ablation of focal atrial fibrillation

Since the early part of this century, the role of focal sources of automaticity in sustaining AF was deemed significant. The application of aconitine to the right atrial appendage resulting in the generation of AF and its ligation resulting in restoration of sinus rhythm was amongst the earliest experimental models for this arrhythmia. Degeneration of rapid atrial tachycardia into AF or onset of AF following multiple atrial ectopic beats offers evidence for this hypothesis. The entity flutter-fibrillation may also possibly result from disorganization of regular atrial activity. However, the pulmonary veins were not clearly implicated in the pathogenesis.

The hypothesis that pulmonary veins and especially the pulmonary vein-left atrial junction as a focal source of AF is supported by anatomical and pathology studies. In 1966, Nathan and Eliakim reported that the proximal portion of the PV has a sleeve of myocardium that is a direct extension from the adjacent atrial tissue and that is electrically coupled to the atrium in an anatomic study of the left atrium-pulmonary vein junction in human hearts [27]. Cabrera et al. studied pulmonary venous anatomy using high-frequency intravascular ultrasound (IVUS) [28]. They obtained cross-sectional IVUS images with a 3.2F, 30-MHz ultrasound catheter at intervals on each vein. Histological cross-sections were compared with ultrasonic images. The pulmonary venous wall at the venoatrial junction revealed a 3-layered ultrasonic pattern consisting of inner echogenic layer representing both endothelium and connective tissue of the media, the middle hypoechogenic stratum corresponded to the sleeves of left atrial myocardium surrounding the external aspect of the venous media and an outer echodense layer of fibro-fatty adventitial tissue. The middle layer was thickest at the venoatrial junction and decreased toward the lung hilum. They found close agreement among the IVUS and histological measurements for maximal luminal diameter and maximal muscular thickness. Ho et al. explored the characteristics of normal pulmonary veins so as to provide more information relevant to radiofrequency ablation [29]. They grossly examined 20 structurally normal heart specimens. Histological sections were made from 65 pulmonary veins. In this study, the longest myocardial sleeves were found in the superior veins. The sleeves were thickest at the venoatrial junction in the left superior pulmonary veins. For the superior veins, the sleeves were thickest along the inferior walls and thinnest superiorly and composed mainly of circularly or spirally oriented bundles of myocytes with additional bundles that were longitudinally or obliquely oriented, sometimes forming mesh-like arrangements. Fibrotic changes estimated at between 5% and 70% across three transverse sections were seen in 17 veins. The longest myocardial sleeves in the superior pulmonary veins in this study correlated with the observation reported by Haissaguerre et al. that the commonest source of the ectopic beats triggering AF were in these veins [12]. The sites mapped during electrophysiology study were 2-4 cm inside the veins, which is longer than the extent of sleeves found in the specimens. These differences were explained as probably due to the indiscrete nature of the venoatrial junction in the specimens, and fixation causing shrinkage of tissues, thus reducing the dimensions. Saito et al. studied thirty-nine human autopsy hearts; 22 with AF and 17 without atrial arrhythmias with intent to provide a detailed anatomy of the extensions of left atrial myocardium onto the pulmonary veins [30]. They observed that the peripheral zones of myocardial sleeves were associated with increasing connective tissue deposition between myocardial muscle groups and suggested that this was a degenerative change that, from the histologic viewpoint, fitted with progressive ischemia. They also suggested that these changes could provide a basis for microreentry and atrial arrhythmias. Lin et al. studied the structure of the pulmonary veins in patients with paroxysmal AF that is initiated by PV ectopic beats [31]. They reported nonspecific dilatation of the ostia and proximal portion of superior pulmonary veins in these patients. They found that the superior PVs were significantly dilated and were the commonest source of the ectopic beats initiating paroxysmal AF. However, site-specific dilatation of the PVs was not evident. Although there was simultaneous dilatation of the PVs and LA, the nonlinear relationship between PV diameter and LA size suggested that the extent of the dilation of the LA and PV ostia was different; it was proposed to be due to the different compliance of the PV vascular wall and LA wall among individuals. The electrical activity in the PVs was presumed to be a result of this extension of cardiac musculature. It is possible that dyssynchronous contraction of the muscle at the atriopulmonary venous junction during rapid and chaotic firing of the ectopic focus may account for an increase in the dimensions of the atriopulmonary venous junction. This study suggested that the ectopic beats might be initiated by the stretch mechanism. Satoh et al. demonstrated that increased atrial stretch in dogs might induce triggered activity resulting in arrhythmia [32]. This dilatation of the pulmonary vein ostia has implications for catheter ablation techniques. An embryological explanation has also been provided for the origin of ectopic foci. Blom et al. studied the development of the cardiac conduction system with the use of HNK-1 immunohistochemistry in human embryos ranging in age from 42 to 54 days of gestation [33]. They hypothesized that in patients with abnormal atrial automaticity, the ectopic pacemaker sites correspond to areas of embryonic myocardium with an early phenotypic differentiation, as indicated by differences in antigen expression during normal cardiac development.

In 1994, Konings et al. classified AF based on the patterns of atrial activation during AF [34]. The Type I AF in their classification, single broad wave fronts propagated uniformly across the RA. This suggests a possible focal origin of AF. In 1997, Jais et al. reported nine patients with paroxysmal focal AF [35]. All were free of structural heart disease and had frequent episodes of AF despite the use of antiarrhythmic drugs. AF was associated with runs of irregular atrial tachycardia or monomorphic extrasystoles. Electrophysiological study demonstrated that all the atrial arrhythmias were due to the same focus firing irregularly and exhibiting a consistent and centrifugal pattern of activation. Three foci were found to be located in the right atrium, two near the sinus node and one in the ostium of the coronary sinus. Six others were located in the left atrium at the ostium of the right pulmonary veins and at the ostium of the left superior pulmonary vein. Haissaguerre et al. in 1998 demonstrated that the pulmonary veins are an important source of spontaneous ectopic beats, initiating frequent paroxysms of AF [12]. The study population consisted of 45 consecutive patients with AF resistant to more than two drugs, at least one episode of AF every two days, receiving anticoagulant treatment, and frequent isolated atrial ectopic beats (more than 700 per 24 hours). Antiarrhythmic medications were discontinued before hospitalization. In 37 patients, at least one episode of sustained AF initiation lasting more than one minute was documented: the ectopic beat initiating AF had a short coupling interval (a P-on-T pattern) and morphologic features similar to those of isolated ectopic beats. Their likely identical origin was confirmed later by intracardiac mapping data. Three multielectrode catheters were placed for the electrophysiology study: one quadripolar roving ablation catheter with a thermocouple, one in the right atrial appendage (to map right atrial and right-pulmonary-vein foci) or coronary sinus (for left-pulmonary-vein foci) to provide stable reference electrograms during mapping, and one for stimulation. If the arrhythmia did not spontaneously develop during electrophysiologic study or was not sufficiently sustained, physiologic procedures (e.g., Valsalva's maneuver or carotid-sinus massage), atrial pacing, pharmacologic agents or all three methods were tried.  The preliminary study involved mapping of isolated ectopic beats. The ectopic focus was localized at the earliest atrial activity relative to the reference electrogram or the onset of the ectopic P wave on the surface ECG. Mechanically produced beats were excluded from the analysis by comparing the electrocardiographic pattern and intracardiac sequence with the confirmed spontaneous ectopic beats. If no sharp bipolar right atrial activity was recorded less than 10 ms before the onset of the ectopic P wave, the beats were considered to have originated in the left atrium. Direct mapping of the left atrium and pulmonary veins was then performed. The role of ectopic beats in the initiation of AF was confirmed by on-site recording of a paroxysm of AF. Ablation was performed at the site with the earliest recorded ectopic activity. Telemetry and 24-hour Holter monitoring were performed to identify the cumulative duration of AF and its frequency. Patients were discharged and given oral anticoagulants for at least three months but no antiarrhythmic drugs were prescribed. Late follow-up consisted of out-patient visits and Holter recordings every three months. A single origin of ectopic beats was identified in 29 patients, two in 9 patients, three in 6 patients, and four in 1 patient. Ectopic beats originated in atrial muscle in 4 patients (in the right atrium in 3 and the posterior left atrium in 1) and in the pulmonary veins in 41 patients [94 percent]): 31 foci in the left superior, 17 in the right superior, 11 in the left inferior, and 6 in the right inferior pulmonary vein. The venous origin of the earliest ectopic activity was demonstrated in 23 patients by the radiographic position of the mapping catheter, which was confirmed by angiographic visualization. The earliest local activity was traced to a point 2 to 4 cm within the main pulmonary vein or one of its proximal branches as evidenced by earliest activation deep in the vein and progressively later toward the ostium and the left atrial exit, resulting in distal-to-proximal venous activation during multipolar recordings. AF was initiated by a single focal discharge in 3 patients, short burst of two or more repetitive focal discharges in 40, and both mechanisms in 2 patients. During sinus rhythm, the activation sequence occurred first in the left atrium and progressively later inside the vein, which was the opposite of the sequence during ectopy. Successful ablation of ectopic foci in the hospital was achieved in 38 patients. Pappone et al. developed an anatomic approach aimed at isolating each PV from the left atrium (LA) by circumferential radiofrequency (RF) lesions around their ostia [36]. They selected 26 patients with resistant AF, either paroxysmal or permanent. A nonfluoroscopic mapping system was used to generate 3D-electroanatomic LA maps and deliver RF energy. Two maps were acquired during coronary sinus and right atrial pacing to validate the lateral and septal PV lesions, respectively. Patients were followed up closely for 6 months. Among 14 patients in AF at the beginning of the procedure, 64% had sinus rhythm restoration during ablation. PV isolation was demonstrated in 76% of 104 PVs treated by low peak-to-peak electrogram amplitude (0.08±0.02 mV) inside the circular line and by disparity in activation times across the lesion. After 9±3 months, 22 patients (85%) were AF-free, including 62% not taking and 23% taking antiarrhythmic drugs, with no difference (P=NS) between paroxysmal and permanent AF.

However, despite all the technological advances, the technique is still far from perfect and is fraught with complications. Ablation of the pulmonary vein focus for AF is a long-lasting procedure with possible complications, such as pulmonary vein stenosis, thromboembolism, air embolism, hemopericardium, and possible damage to such adjacent structures such as bronchioles, the right pulmonary artery, and lung tissue. Paroxysmal AF can also recur. According to Wellens, the following are the problems with the current technique [37]. First, the ectopic foci, most commonly found in the superior pulmonary veins and are characterized by a sharp electrogram, which may or may not be conducted to the atrium. A rapidly firing focus may be the mechanism for paroxysmal AF, but 1 or 2 pulmonary vein ectopic beats may also initiate AF in the presence of an additional substrate in the left or right atrium. These potentials have to be differentiated from those of the ligament of Marshall and the right atrium, which can also be foci of paroxysmal AF. Accurate mapping is helped by use of multielectrode catheters or electroanatomic mapping systems and will hopefully be facilitated by clear demonstration of the premature P wave polarity. Unfortunately, ectopic pulmonary vein ectopy may not be present during the electrophysiological study, even with provocative pharmacological or pacing procedures. The second problem is about the energy source for ablation. Currently, most experience is with heat with radiofrequency (RF) energy. Studies are ongoing on the appropriate RF power, heat limits, pulsed versus continuous RF energy delivery, and the use of irrigated-tip RF ablation. This has led to the development of ultrasound delivered through a balloon in the pulmonary vein, laser, and cryoablation. More knowledge is needed about the histopathological consequences of these approaches. Third, much activity is going on in the development of the appropriate shape of the catheter at the site where the energy has to be delivered. The purpose of ablation is to eliminate the high-frequency pulmonary vein potential and the creation of bidirectional block in the pulmonary vein. To prevent ectopic pulmonary vein activity from entering the left atrium, a circular lesion in or around the orifice of the pulmonary vein seems a logical approach. Obviously, technical developments have to occur to speed up localization and isolation or eradication of ectopic activity. The last problem concerns the long-term results of pulmonary vein ablation in AF. The short-term results of pulmonary vein ablation are promising but uncertainty exists about long-term results with regard to arrhythmia recurrence and mechanical complications such as development of pulmonary vein stenosis. Also, the best way to evaluate the possible development of pulmonary vein stenosis needs to be established: echocardiography, CT, MRI, or angiography.  There also remains the question of reversibility of electrophysiological changes in the atrium induced by AF (“electrical remodeling”). Wijffels et al. have demonstrated this phenomenon in the goat heart and this has been confirmed by other investigators in the human heart [38]. It is unknown up to what time point in the natural history of AF those changes are still reversible and the patient can be helped by removal of the initiating trigger.

The coronary sinus has also been suggested to have a role in the origin and sustenance of AF. Antz et al. studied the presence of electrical connections between the coronary sinus musculature and the right and left atrial myocardium, forming a RA-LA connection [39]. Gerlis et al. reported two cases of ventricular pre-excitation due to accessory pathways histologically identified as being associated with coronary sinus aneurysms [40]. They noted that the proximal coronary sinus is surrounded with a spiral myocardial sheath that stops abruptly at or shortly beyond the orifices of the entering coronary veins, including the great cardiac vein, and is continuous with that of the morphological right atrium. This is a remnant of sinus venosus musculature. Recently, Katritsis et al. recorded double potentials within the CS, particularly the distal superoposterior part, near the left superior pulmonary vein [41]. The prevalence was higher in patients with PAF than in subjects with other or no arrhythmia. Hence, they proposed that their presence denotes possible sources or substrate for atrial arrhythmia.

The ligament of Marshall is another focus of AF that has been described in the literature. It consists of multiple sympathetic nerve fibers, ganglia, blood vessels and multiple myocardial tracts (Marshall Bundles) insulated by fibrofatty tissue. Kim et al. studied seven hearts at postmortem to study the anatomy of the ligament of Marshall [42]. They concluded that the ligament of Marshall in human hearts is innervated by sympathetic nerve fibers and has multiple myocardial tract insertions into the left atrial free wall and CS, forming a substrate of reentry. Polymeropoulos et al recently reported a 66-year-old woman with a history of typical atrial flutter and atrial fibrillation in whom endocardial recordings during tachycardia from this region showed a discrete electrical potential (Marshall potential) preceding the atrial electrogram [43]. Radiofrequency ablation was successfully performed.

### Palliative radiofrequency procedures for AF

Permanent AF is usually not amenable to ablation procedures and may require multiple pharmacological agents for management. In some of these patients, medical therapy is poorly tolerated or unsuccessful. In these cases, atrioventricular node (AV node) ablation combined with permanent pacemaker implantation is a possible treatment strategy with rate control as the goal. The pacemaker may be single chamber (VVI) for chronic AF or dual chamber (DDD) for paroxysmal AF. This procedure is successful in almost 100% cases and late recovery of AV conduction is rare. However, this procedure still mandates anticoagulation and possibly requires anti-arrhythmic therapy. Olgin and Scheinman compared high-energy direct current and radiofrequency catheter ablation for ablation of the atrioventricular junction [44]. They concluded that radiofrequency energy is as efficacious as and safer then high energy current. Radiofrequency ablation of the AV junction may be performed using either a left or right-sided approach. Kalbfleisch et al. prospectively compared the left sided approach with persistent attempts from the right side in patients in whom initial radiofrequency applications on the right side were unsuccessful [45]. The left sided approach required significantly fewer radiofrequency applications after randomization than the right-sided approach. The Ablate and Pace Trial  studied the prevalence and characteristics of escape rhythms after radiofrequency ablation of the AV junction [46]. This was a prospective, multicenter registry of AF patients undergoing AV node ablation and pacing. Before discharge from the hospital, the patients underwent a systematic analysis of the rate and morphologic features of the escape rhythm, if any that was present when the pacing rate was gradually decreased. This study reported that majority of the patients who undergo radiofrequency ablation of the AV node are pacemaker dependent after the procedure, as defined by lack of an escape rhythm that is < 40 bpm. Reports of ventricular arrhythmias and sudden death after ablation have raised concerns about safety. Polymorphic ventricular arrhythmias are related to electrical instability due to an initial prolongation and then slow adaptation of repolarization caused by change in the heart rate and activation sequence. These stabilize during the first week after the procedure. Routine pacing at 80 bpm during this phase is recommended as well as in hospital monitoring for at least 48 hours. Patients with high risk features for arrhythmias such as congestive heart failure or impaired left ventricular function, may require pacing at higher rates. Adjustment of the pacing rate, although rarely below 70 bpm is usually undertaken after a week in most patients, preferably after an electrocardiographic evaluation for repolarization abnormalities at the lower rate [47].

AV node modification has been attempted with the intent to obviate the need for permanent pacemaker implantation. AV node modification can be done by the anterior or posterior approach. To compare the safety and efficacy of the anterior versus the posterior approach for AV node modification, Lee et al. randomly assigned 40 patients with medically refractory paroxysmal AF to receive AV junction modification with an anterior or posterior approach [48]. They concluded that (1) the two techniques had similar efficacies, (2) if one approach was ineffective, switching to the other approach might be safe, (3) combining these two approaches resulted in overall improvement in the success rate of this procedure, and (4) the posterior approach needed more radiofrequency pulses, longer procedural and fluoroscopy exposure time. Rokas et al. evaluated the role of RR interval distribution pattern as an outcome predictor of radiofrequency modification of the AV node in chronic AF and the likely mechanism of rate control [49]. The RR interval distribution pattern was measured from 24-hour ECG recordings obtained before and after the procedure. The preablation pattern was described as bimodal (B) or unimodal (U). Bimodality was defined as existence of 2 RR populations separated distinctly by a visually estimated intersection point, the value of which must be the same in >2 consecutive heart rate measurements. This criterion was described earlier by the same investigators in an earlier study using RR interval distribution analysis as a noninvasive method for detecting dual AV nodal physiology. They concluded that AV node modification is expected to be more effective, safe and expeditious in patients with chronic AF and a ‘B’ type RR interval distribution pattern whereas ‘U’ pattern patients may benefit from partial injury to the AV node and that posterior atrionodal input ablation may be the mechanism for rate control in these patients. Lee et al. compared the long-term effects of complete AV junction ablation with those of AV junction modification in patients with medically refractory AF [50]. They concluded that AV junction ablation with permanent pacing had a significantly greater ability to decrease the frequency of attacks and the extent of symptoms of AF, and more satisfaction with their general well-being among these patients.

Transcatheter radiofrequency AV node ablation followed by ventricular pacing has been shown to improve symptoms and quality of life of the patients with AF. Twidale et al. reported a modest and progressive improvement in cardiac performance following improved control of rapid heart rate after successful AV node ablation [51]. Edner et al. reported that in patients with left ventricular dysfunction long term improvement of systolic and diastolic left ventricular function was seen after ablation of the atrioventricular junction for rate control of atrial fibrillation [52]. This procedure had no adverse effects on normal left ventricular function. Natale et al. showed that a chronic irregular heart rate alone could produce an overall reduction in cardiac function that can be reversed by AV node ablation and pacemaker implant [53]. Therefore, ablation of the AV junction and permanent pacing could represent a more appropriate therapeutic modality over procedures or treatments targeting rate control, particularly in patients with left ventricular dysfunction. Brown et al. hypothesized that the observed improvement in ejection fraction with control of the persistently rapid ventricular response to AF would account for the improved functional class in these patients [54]. They also hypothesized that heart rate control would lead to an improvement in papillary muscle function, with a relation between a reduction in mitral regurgitation and improved functional class. They reported 15 patients with atrial fibrillation and severe heart failure who had marked functional improvement after AV nodal ablation with VVIR pacemaker implantation. According to their study, although the ejection fraction improved in 53% and mitral regurgitation decreased in 57 %, the improvement could not be explained entirely by either a change in ejection fraction or reduction of mitral regurgitation. However, in a recent study to assess the long-term effect of AV node ablation and ventricular pacing on left ventricular ejection fraction (LVEF) in patients with permanent AF, Szili-Torok et al. concluded that restoration of a regular ventricular rhythm following AV node ablation for patients in permanent AF does not result in improvement in left ventricular function [55]. It is worthwhile considering these palliative approaches in patients with chronic AF with rapid ventricular rates where drug therapy is not tolerated or unsuccessful. Possible improvement in left ventricular function following AV node ablation also makes this a consideration to subjecting the patients to toxic and potentially proarrhythmic pharmacological therapy.

## Summary

From the above review, it is evident that strides in evolution of intrventional therapies for AF have been extensively guided by improved understanding of pathophysiology, electrical basis, mapping and catheter technology. The question of the electrophysiological mechanism   of AF continues to haunt us: re-entry or automaticity? Probably both. The mechanism may have to be deciphered on an individual case basis. Valuable clues can be obtained by looking at multiple temporally separated ECG strips (conventional 12 lead ECG, Holter monitor or event monitor) from patients with AF which can reveal frequent unifocal or multifocal atrial ectopy initiating AF, or AF triggered by sustained supraventricular tachycardia or atrial flutter amidst AF. Other clues may surface during electrophysiology study by analysis of intracardiac ECGs, especially the mechanism of onset of AF. Although some of the sites described as focal AF triggers are only anecdotal reports, they are important to consider in an individual patient with AF. These reports also make it possible that other yet unidentified areas are AF triggers. It is also important to determine whether these foci are producing AF by themselves or merely helping sustain it. Further advances in mapping, localization and ablation techniques and technology will determine the role of these foci and pave the way for curative radiofrequency ablation of AF.
